# “Running myself ragged”: stressors faced by peer workers in overdose response settings

**DOI:** 10.1186/s12954-020-00449-1

**Published:** 2021-02-11

**Authors:** Zahra Mamdani, Sophie McKenzie, Bernadette Pauly, Fred Cameron, Jennifer Conway-Brown, Denice Edwards, Amy Howell, Tracy Scott, Ryan Seguin, Peter Woodrow, Jane A. Buxton

**Affiliations:** 1 BC Centre for Disease Control, 655 West 12th Avenue, Vancouver, BC V5Z 4R4 Canada; 2 Canadian Institute for Substance Use Research, STN CSC, University of Victoria, Box 1700, Victoria, BC Canada; 3 SOLID Outreach Society, 1056 N Park St, Victoria, BC V8T 1C6 Canada; 4 RainCity Housing, 616 Powell St, Vancouver, BC V6A 1H4 Canada; 5 School of Population and Public Health, University of British Columbia, 2329 West Mall, Vancouver, BC V6T 1Z4 Canada

**Keywords:** Peer workers, Peers, Overdose, Harm reduction, Stressors, Stigma

## Abstract

**Background:**

Peer workers or “peers” (workers with past or present drug use experience) are at the forefront of overdose response initiatives, and their role is essential in creating safe spaces for people who use drugs (PWUD). Working in overdose response settings has benefits for peer workers but is also stressful, with lasting emotional and mental health effects. Yet, little is known about the stressors peer workers face and what interventions can be implemented to support them in their roles.

**Methods:**

This project used a community-based sequential mixed-methods research design. Eight peer researcher-led focus groups (*n* = 31) were conducted between November 2018 and March 2019 to assess needs of peer workers. The transcripts were thematically coded and analysed using interpretative description. These results informed a survey, which was conducted (*n* = 50) in September 2019 to acquire quantitative data on peer workers’ perception of health, quality of life, working conditions and stressors. Frequency distributions were used to describe characteristics of participants. *X*^2^ distribution values with Yates correction were conducted to check for association between variables.

**Results:**

Five themes emerged from the focus groups that point to stressors felt by peer workers: (1) financial insecurity; (2) lack of respect and recognition at work; (3) housing challenges; (4) inability to access and/or refer individuals to resources; and (5) constant exposure to death and trauma. Consistent with this, the factors that survey participants picked as one of their “top three stressors” included financial situation, work situation, and housing challenges.

**Conclusion:**

Peer workers are faced with a diversity of stressors in their lives which often reflect societal stigmatization of drug use. Recognition of these systemic stressors is critical in designing interventions to ease the emotional, physical and financial burden faced by peer workers.

## Background

Several regions of the world, including Europe and North America, are amid a devastating drug overdose epidemic [1–3]. In Canada, more than 15,393 individuals have died because of an apparent opioid-related overdose between January 2016 and December 2019 [4]. Due to the escalating rate of overdose deaths, the British Columbia (BC)  Provincial Health Officer declared a public health emergency in April 2016 which is still in effect today [5]. On March 17, 2020, a second public health emergency related to the pandemic of the coronavirus disease (COVID-19) was declared [6].

Evidence from the BC Coroners Service shows that illicit drug toxicity deaths have been on the rise since the onset of COVID-19 and the implementation of physical distancing measures in March 2020 [7]. In July 2020, there were 175 suspected illicit drug toxicity deaths [7]. This represents the third consecutive month where the number of illicit drug toxicity deaths has surpassed 161, the highest number previously recorded in a month in BC [7].

In BC, peer workers, often referred to as “peers”, are at the forefront of overdose response initiatives [8, 9]. In this context, peer workers are those with past or present drug use experience who use that lived/living experience to inform their professional work [10]. Peer workers inform and lead nimble and effective overdose response and prevention services for people who use drugs (PWUD) [11–14] within overdose response settings.^1^ These include stand-alone supervised consumption sites and overdose prevention services (OPSs), as well as services in shelter and housing agencies. The advent of COVID-19 has led to reduced hours and closure of several OPSs [15, 16]. This has further increased the importance of peer workers who are involved in a variety of roles, including peer-witnessing onsite, outreach services, mobile overdose response, delivery and collection of harm reduction supplies, advocacy, and referrals to services such as housing agencies [17].

A growing body of evidence indicates that peer worker-led programs are successful in creating “safe spaces” for PWUD [12, 14, 18–21] and help to reduce harmful health behaviours such as sharing substance use supplies and unsafe sex practices [22, 23]. Peer-led programs also improve program accessibility and acceptability [10], help in building connections and trust [24], facilitate environments of comfort and safety for service users [12, 13], and are associated with mental health benefits for PWUD [25]. Accumulating evidence suggests that individuals with lived/living experience of substance use are leading the harm reduction movement in meaningful ways, successfully reducing the harms associated with drug use and structural violence [12, 13, 20, 26, 27].

Despite the benefits of peer-led harm reduction programming, several studies have shown that working in overdose response settings can be stressful and traumatizing, with lasting social, emotional and mental health effects for individuals [12, 19, 26, 28–30]. Individuals working in overdose response face grief due to the significant loss of lives during the overdose epidemic [12, 19, 26, 30]. A recent study indicated that even a single exposure to a fatal or non-fatal overdose can lead to considerable stress, burnout, and overdose-related compassion fatigue [31].

There are benefits and challenges associated with work for peer workers in overdose response settings [24]. While work can provide peer workers with a sense of purpose and an opportunity to inspire others and contribute to their community, exposure to ongoing loss and trauma may be particularly stressful, as the individuals they support are often close friends or people they consider family [24]. Peer workers not only *work* in a stressful environment, but often *live* the same reality too [32–34]. Like other PWUD, peer workers are in a vulnerable position, subject to societal stigma, poverty, poor living conditions, and illness [35–37]. These experiences of oppression that harm and constrain individuals or groups based upon their social positioning are termed “structural vulnerability” [38], and these conditions can be considered violent in that they do harm and contribute to poor health outcomes.

Several studies have found that individuals without lived/living experience of substance use often have negative attitudes towards PWUD [39–41]. These stigmatizing attitudes permeate into workplaces and lead to inequity and differential treatment of peer workers. Further, other researchers have found that peer workers often receive minimal financial compensation for their work, have precarious work arrangements and lack job security [12, 42]. This, coupled with the stress of the work itself can contribute to burnout [12, 20].

While other front-line workers engaged in overdose response (i.e. paramedics, nurses, or other first responders) have access to counselling, training and support through their employers [43, 44], peer workers often lack such supports. Given the benefits as well as growing utilization of peer workers in harm reduction efforts, it is important to understand the stressors faced by peer workers and design holistic support interventions to ameliorate the negative emotional and mental health effects of working in these stressful environments.

The overall aim of our research project, titled Peer-2-Peer Project, is to identify and implement supports designed for and by peer workers in overdose response settings. In this paper, we have highlighted the stressors faced by peer workers, both within their work environment and in their daily lives. These insights will lay the foundation to identify and implement suitable supports developed for and by peer workers.

## Methods

The research was based at two organizations, located in four urban cities that spanned three of the five BC health regions: (1) Solid Outreach—a peer worker-led organization in Vancouver Island that educates, advocates and provides services for individuals that use substances [45], and (2) RainCity Housing—a not-for-profit, housing-first organization in Vancouver Coastal and Fraser regions that provides housing and support services for people living with mental health, substance use and other challenges [46].

This project used a community-based research design, and the research team consists of both academic researchers and community-based peer research assistants (PRAs). The PRAs were people with lived/living experience of substance use who were recruited by managers of the pilot organizations and trained in research methods. Initially, five PRAs were recruited; more PRAs were added to the team as several PRAs had to leave at various points in the project due to competing priorities or personal circumstances.

A sequential mixed-methods design was used for data collection. During the first phase of data collection, peer workers were invited to participate in qualitative focus groups in four cities. The results of the focus group informed the development of the quantitative survey, which was administered to peer workers during the second phase of data collection.

### Phase 1: Qualitative focus groups

Focus groups were conducted between November 2018 and March 2019. Purposeful sampling (by PRAs or organizational managers) was used to identify and recruit participants with in-depth knowledge about peer work from different positions, circumstances and perspectives [47, 48]. The inclusion criteria for participants were: (1) working, formally or informally, in overdose response settings, (2) identifying as a peer worker, (3) being over the age of 18, (4) being able to complete a brief demographic questionnaire and a focus group interview in English.

The purpose of the focus groups was to identify the beneficial aspects as well as the stressors and challenges faced by peer workers in their jobs, and explore their needs for support. The beneficial aspects of their work are reported elsewhere [24]. Conversations were directed by a semi-structured focus group guide, which was developed based on the research objectives, preliminary discussions with PRAs and literature review. Focus groups started with a brief description of the project and the goals of the focus group. Informed consent was then obtained, and participants were asked to complete a one-page demographic questionnaire. The focus groups were conducted by two members of the research team: a PRA who facilitated the focus group, and an academic researcher who took field notes and provided logistical and methodological support.

In total, eight focus groups were conducted; two in the Fraser region (one in Coquitlam, one in Maple Ridge), two in Vancouver Coastal and four in Island Health (Victoria). Each focus group had three to six participants; the groups were intentionally small to allow participants enough time to voice their thoughts within one hour. A total of 31 peer workers participated in the focus groups. The demographic profile of these participants is presented in Table 1. Answering any question on the demographic questionnaire was optional, and some participants opted to leave a few questions blank. Just over half of all the focus group participants were male (55%). Of those that completed the questions related to age and education (*n* = 18, 58% of total), 56% were over the age of 40 and all reported having received at least some high school education.

**Table 1:** Demographic characteristics of focus group participants (*n* = 31)

	*n* (%)
*Gender*	
Male	17 (55%)
Female	14 (45%)
*Age*	
40 and under	8 (26%)
41–50	4 (13%)
51+	6 (19%)
Unknown	13 (42%)
*Highest level of education*	
No schooling	0 (0%)
Some elementary school	0 (0%)
Some high school	10 (33%)
Some comm. college/tech school/street degree/university/ post grad. training	8 (24%)
Unknown	13 (42%)

Each focus group lasted approximately one hour. Focus groups were audio-taped, and each participant received food and $25 CAD in cash, as per the BC peer worker payment standards [49]. The audio recordings were transcribed verbatim by an external transcriber, and the transcripts and field notes were de-identified. Demographic identifiers are not associated with quotes to protect peer workers from being recognized.

We used thematic analysis to identify, organize, and report themes, which aligned with the applied focus of our study [50, 51]. A participatory coding process was used to sort the quotes into relevant codes that helped surface the underlying meanings behind the quotes [52]. A coding framework was developed with input from PRAs and inputted into NVivo (QSR International, version 12) where data analysis progressed in an iterative and reflexive manner. We specifically drew on interpretive description to generate practical and applied knowledge from the data, situating findings within the real-world context [52]. This was done with the help of the PRAs who classified quotes into themes and sub-themes and determined hierarchies. PRAs were also responsible for selecting quotes most reflective of their experience for presentation within this paper. The PRAs and academic researchers on our team have made every effort to select quotes that represent views of all four sites. Key stressors and intervention needs were identified by PRAs, and a model comprising of these priorities was developed collaboratively [53].

### Phase 2: Quantitative survey

The second phase of data collection was the administration of a quantitative survey by PRAs to peer workers in the four cities where pilot sites are located in September 2019. While the primary aim of the survey was to obtain a baseline snapshot of peer workers’ perspectives prior to the implementation of interventions, it also assisted in gaining a more comprehensive understanding of the stressors faced by peer workers and the supports they need. The survey was informed by the focus group findings and consisted of demographic questions, measures of peer workers’ perceptions of health and quality of life, substance use patterns and working conditions. The majority of the survey questions were adapted from validated tools with good psychometric properties, including the Canadian Community Health Survey [54], Short Form-12 (SF-12) Health survey [55], the Professional Quality of Life Scale (ProQOL) [56], and the Job Satisfaction Survey [57].

The survey was completed by 50 peer workers: 17 from the Fraser region (nine from Coquitlam, eight from Maple Ridge), 16 from the Vancouver Coastal region and 17 from the Island Health region (Victoria). The demographic profile of survey participants is presented in Table 2. Similar to the demographic profile of focus group participants, the majority of the survey participants were male (54%), over 40 years old (58%), and had obtained at least some high school education (94%). Also, most survey participants self-identified as non-Indigenous (68%).

**Table 2:** Demographic characteristics of survey participants (*n* = 50)

	*n* (%)
*Gender*	
Male	27 (54%)
Female	22 (44%)
Other	1 (2%)
*Age*	
40 and under	19 (38%)
41–50	20 (40%)
51+	9 (18%)
Unknown	2 (4%)
*Ethnicity*	
Reported indigenous	16 (32%)
Non-indigenous	34 (68%)
White	32 (64%)
Black/Latin American	1 (2%)
Other	1 (2%)
*Highest level of education*	
No schooling	0 (0%)
Some elementary school	3 (6%)
Some high school	28 (56%)
Some comm. college/tech school/street degree/university	19 (38%)

The analysis of quantitative survey data was conducted by an academic researcher and summarized in a report which was presented to the PRAs for data validation. In this paper, we provide insight into stressors faced by peer workers utilizing participant responses to the question “Thinking about stress in your day-to-day life, what would you say are the *top three* most important things contributing to feelings of stress you may have?”. Frequency distributions of the three factors selected as “top three stressors” are presented. Pearson Chi-square tests for independence were conducted for gender, age, location, and ethnicity comparisons for the different factors selected as stressors. Yates correction was used to adjust for the smaller sample size and low cell count values. Data were analysed using R statistical software, version 4.0.2 [58].

In addition to conducting data validation with the PRAs, findings from both the qualitative and quantitative components of the research were shared with representatives from each of the three health authorities where the pilot sites are located as well as groups who have expertise in the environments where peer workers are employed.

## Results

### Stressors and challenges faced by peer workers

Several sub-themes of stressors and challenges in peer work were identified by focus group participants. These themes and sub-themes are summarized in Table 3 and presented sequentially in more detail below.

**Table 3:** Summary of stressors reported by peer workers during focus groups

Stressor	Sub-themes
Financial insecurity	Inequitable pay Job instability
Lack of respect and recognition at work	Stigma and inequity Lack of resources to professionalize peer worker roles Lack of role clarity Lack of respect and recognition at work from colleagues Lack of respect from other professionals
Housing challenges	Living situation jeopardizes safety and health Difficulty in acquiring housing due to stigma against PWUD Safe housing is unaffordable Affects peer workers’ productivity
Inability to access or refer individuals to resources	Lack of social supports to access needed resources Inability to refer clients to resources leading to a sense of powerlessness and dissatisfaction Stigma and judgement affecting access of existing resources
Constant exposure to death and trauma	Working as a first responder can cause stress and burnout Constant exposure to death and trauma is doubly stressful for peer workers, especially death of friends and family Personal and professional lives are inter-twined; no opportunity to unwind

In cases where the survey data for a particular theme from the focus groups were available, they are presented below (see Tables 4 and 5 in appendix).

### 1. “Scrape up money… to just get by”: Financial insecurity

Poor financial situation of workers was consistently mentioned by participants as a key stressor. As one participant stated: *“*Honestly, my biggest hurdle is trying to do my job and then try and scrape up money outside of work to just get by”. This sentiment reflects the severe financial hardships and often “hand-to-mouth” situation many peer workers are faced with.

Similar to focus group participants, survey participants also indicated financial insecurity as a key stressor. When asked to choose from a list of 16 potential stressors (see Appendix), overall 72% of participants listed financial situation as one of their top three stressors (Table 4). This perception varied between the four cities (ranging between 62.5% and 88.9%), however the difference was not significant *p* = 0.71. There were also no statistically significant differences in the perception of financial situation as a one of the top three stressors across the different age groups, genders and ethnicities.

#### Inequitable pay

Several focus group participants attributed their financial insecurity to the inequitable pay they received. Participants spoke about getting paid much less than other support staff employed by their organizations, despite doing the same work. This is apparent in one participant’s words: “[Support workers] are making one amount. [Peer workers] are making another, doing the exact same job”. This highlights the inequities between staff doing the same kind of work.

Some participants suggested that the pay inequity stems from the higher value that organizations place on formal education and certification over expertise acquired though lived/ living experience. As one participant mentioned: “We’ve got people that […] took an eight-month course […] with two months of addictions training and somehow that certification is valued above lived experience”. This quote illustrates that there is a lack of recognition of the value of lived/ living expertise as an important source of knowledge.

The idea of pay inequity was also echoed in survey responses: When asked if individuals feel they get paid a fair amount, 45% of the participants disagreed and 14% were neutral (Table 5). The proportion of participants that disagreed ranged between 14 and 69% in the four cities.

These findings suggest that ongoing pay inequities contribute to the financial insecurity faced by peer workers. The sub-theme of inequitable pay is suggestive of a larger issue related to lack of respect and recognition of lived/living expertise, which is discussed as a separate theme below.

#### Job instability

Job instability compounds financial insecurity faced by peer workers. Many peer workers mentioned that their jobs rely on unstable funding and lack formal long-term employment contracts. As one focus group participant described, “There’s only so much money in this ‘well’, because this is government funded. Now if they pay us what they’re paying the people over there, how much sooner is our well going to run dry? When am I unemployed? That scares the hell out of me. […] Am I going to be here next year?”. The use of the metaphor of a “well” indicates that peers work in a precarious environment, often characterized by job instability and lack of job security. The quote also highlights that lack of funds is often used to justify inequitable pay for peer workers and that invokes fear of unemployment, which is a significant stressor for peer workers.

### 2. “Sets us apart”: Lack of respect and recognition at work

One of the top stressors that emerged from the focus group data is the lack of recognition and respect for peer workers. Several peer workers felt that they were not taken seriously or given due respect by their work colleagues and by other professionals they encounter in their work.

Many peer workers expressed that the use of the term “peer” is not an adequate job title as it sets to define the work of peer workers solely by their history of substance use, rather than by the important work they do and the lived/living experience and context they provide. As such, it is considered stigmatizing and derogatory. As one peer worker mentioned, “This term “peer,” I’m really quite uncomfortable with [it]. […] It divides us. […] It sets us apart from normal society”. This quote highlights that term “peer” can be othering and leads to differential treatment of peer workers by others. Thus, the lack of respect seems to stem from stigma against PWUD.

The differential treatment of peer workers is apparent in the lack of basic resources for peers, which are generally provided to most working professionals, such as photograph IDs or business cards. As one peer worker mentioned, “We don’t have the exact same things as the other support staff”.

Focus group participants also pointed out the absence of formal job descriptions and contracts for peer workers, leading to lack of role clarity among supervisors as well as their support worker colleagues. Several focus group participants mentioned that they are often assigned to menial tasks and are looked down upon despite their extensive expertise and skillset. As one participant described:

Sometimes when I get [to work] and I’m barely even in the door [support workers are] like, ‘you gotta go upstairs and go clean the kitchen. You gotta go in here and clean the staff room’.

This quote indicates the constant struggles that peer workers face at work and how they are given minimal respect due to their perceived position in the organization.

Peer workers also expressed that they do not feel respected by other professionals they encounter in their work. One peer worker described a situation that they faced with the police:

Today, for example, a guy was O.D.’ing just across the street here. [We] went over, we had our Narcan kits and we had everything under control. We were doing our job. Next thing you know we got eight cop cars there and they’re telling us we’re going to be arrested if we don’t leave.

This quote shows how police officers and other service providers may not understand the life-saving work peer workers constantly perform which illustrates lack of recognition and respect for their work. A similar sentiment was expressed by another peer worker who recounted their experience with paramedics:

I think there’s a little bit of stigma from the ambulance people [and] from the first responders. […] I find that they can come in, take over and kind of push the peer aside. I think that there is a lot of stigma against peers.

This quote indicates that the lack of respect for peer workers is rooted in deeper societal stigma that uniformly characterizes peer workers by their substance use and is indicative of a broader system built to further marginalize PWUD.

Similar to focus group participants, survey participants feel that they don’t always get the recognition that they deserve for their work. For the question, “when I do a good job, I get the recognition for it that I should receive”, half of the participants (50%) disagreed or were neutral (Table 5). The proportion of participants that disagreed ranged from 12 to 78% in the different cities. Furthermore, more than a third of the survey participants (40%) listed “work situation (including working conditions)” as one of their top three stressors (Table 4). This ranged from 12.5% to 77.8% in the four cities (*p* = 0.03). The differences across genders, ages and ethnicities were not statistically significant.

These results highlight how much importance peer workers place on being recognized and respected at work; lack of respect can be a stressor for them.

### 3. “My shithole”: Housing challenges

Another stressor identified by peer workers was housing challenges which in turn impacts the ability to sustain work. Several participants expressed that they would like to have “somewhere [they] can call a home”. Having a safe place one can call “home” is crucial for peer workers’ productivity and ability to help others. As one participant explained:

When you’re able to look after yourself financially and physically and mentally and emotionally, then you’re able to do so much more for other people. Because you’re together. You’re not worried about that. Stressing out, [worrying] about [how] after this, I got to go home to my shithole and try to figure out what I’m going to do for […] dinner. You’re together and you’re in a position where you can actually help.

Several focus group participants mentioned that their housing situations jeopardized both their security and their health. For example, participants mentioned being assaulted in their buildings, having accommodation infested with bed bugs, and having small quarters that felt like a “jail cell”. For peer workers, the conditions of their homes seem to add stress rather than offer a respite to alleviate it.

Some participants mentioned that they felt helpless and were unable to move because they often encounter problems in acquiring housing. BC is known for its exorbitant housing rental prices [59–61], which makes safe housing unaffordable for peer workers with meagre wages. One participant mentioned: “I can’t move anywhere else. I can’t afford to move.” This quote indicates that many peer workers are forced to endure poor living conditions due to a housing market that favours the wealthy and privileged.

Some peer workers described that they find it difficult to find a home within their communities, surrounded by people that they feel safe with. One participant mentioned: “I don’t want to leave my community. […] I still want to stay in [my community]. It’s where my friends, my family is”.

Like focus group participants, survey participants also listed housing challenges as a common stressor. More than a third (38%) of the survey participants listed housing-related issues as one of their top three stressors (Table 4). Perception of housing situation as one of the top three stressors ranged from 12.5 to 66.7% (*p* = 0.31) in the four communities. Differences across ages, genders and ethnicities were not significant.

When asked if individuals *faced housing challenges due to their substance use in the last 30 days*, almost a half (48%) of the participants indicated that they “always”, “often”, or “sometimes” faced such challenges (Table 5). Although not the majority, 48% is a large percentage of respondents that faced housing challenges in such a short amount of time (30 days). In some cities, almost all participants reported having faced housing challenges in the last 30 days (Table 5). Clearly, acquiring acceptable housing is a challenge for peer workers, and this can add considerable stress to their lives.

### 4. “Not enough social support”: Inability to access or refer individuals to resources

Many focus group participants indicated that a lack of social services for PWUD is a key stressor in their lives. Participants described that they often serve as a bridge connecting PWUD to social services and other supports. Inability to do so, despite a genuine desire to help adds to peer workers’ stress and dissatisfaction. This sentiment is expressed by one participant who mentioned, “I’m not as worried about myself as the clients, but there’s not enough social support… in all aspects”. Several examples of resources which PWUD find hard to access were provided by focus group participants, including “detox”, legal services, welfare and income assistance, and civic services such as government identification.

Some peer workers expressed that certain services do exist, but tend to be inaccessible for PWUD, either directly by prioritization of more privileged groups, or indirectly through stigma and judgement which makes PWUD hesitant to use them due to past negative experiences. Health care is one such resource; many peer workers feel that the stigma against substance use often clouds healthcare providers’ ability to provide compassionate care. As one participant noted, “I feel like a real asshole, like, trying to convince anybody to go to the hospital because I know why they don’t want to go”*.* Albeit subtly, this quote communicates a powerful message; it indicates that peer workers, along with PWUD, often do not want to access health care because of stigma and are left to navigate their own physical and mental health challenges.

In some cases, although resources do exist, peer workers often do not feel equipped with the skills and credibility to make referrals to those services. Sometimes this lack of credibility is due to lack of institutional authority to make referrals. This often leads to a sense of powerlessness adding stress and frustration to peer work. The following quote highlights the desire of a peer worker to be able to do more:

If you want to help somebody you don’t want to turn them away […] without getting an answer or resolving their enquiry. You want to help them and it bothers you ‘cause you’ve been on that side where nobody’s helping you and it’s frustrating.

The powerlessness felt by peer workers is rooted in a point discussed earlier—inequity in the workplace. Peer workers do not have access to the same resources as other staff at work, including skill development opportunities. These findings indicate that inability to refer PWUD to the services they need, either due to lack of those resources or due to lack of skills and credibility to make referrals, causes significant stress for peer workers.

### 5. “Living through our losses”: Constant exposure to death and trauma

Several peer workers mentioned that the constant exposure to trauma and loss of lives is emotionally taxing and stressful. Like other first responders, peer workers can get stressed and burnt out from working in overdose response settings. As one peer worker described, “Being a first responder can drain you after a day, after a few of them”. In addition, unlike most other first responders, peer workers have a unique understanding of the lives of PWUD and can relate deeply to stories of trauma, which can amplify the stress they feel. In this same vein, peer workers are not supporting mere clients but often clients who are friends and family members. Losing a client, therefore, is so much more difficult. As one participant mentioned, “I lost a couple of my best friends in the last couple of years and it’s just been really friggin’ hard”.

For peer workers, personal and professional lives are heavily intertwined because they are part of the community they serve. Unlike other professionals, their work is a 24-h job. Peer workers often do not get to unwind after a stressful day because they are constantly working to support community members in need, even outside the work environment. Peer workers also worry about their friends and members of their community. As one participant stated:

I just get worried about people that I know, like, friends that [are] still doing the same old thing. You like worry about [whether] they [are] going to […] OD or if they’re going to be okay.

This constant worrying and the inability to unwind can eventually take a toll on peer workers’ mental health and well-being. As one peer worker illustrates: “I am running myself ragged. The burnout”.

Peer workers live and work in an environment so often punctuated by loss. This idea is summarized by a focus group participant who mentioned that the most stressful thing for them was:

Living through the bad days. Living through our losses. Living through somebody [going] to jail. Living through somebody [getting] beat[en] up down on the corner and [having] their head bashed in, [spending] six months in the hospital. Living through […] we found a dead body in the garbage. Living through those things together. I think that is a lot. I think that’s it, really.

Consistent with the focus group findings, two-thirds (68%) of the survey participants that responded were at least sometimes “affected by the traumatic stress of those they help”, i.e. the sum of those that stated “always”, “often” and “sometimes” (Table 5). Furthermore, almost half (47%) of the respondents reported that they “feel worn out because of their work” (Table 5). A similar proportion of respondents (47%) reported that they “find it difficult to separate their personal lives from their lives as peer workers” (Table 5).

Despite the traumatic stress and burn-out peer workers report, only slightly over a quarter (28%) of the survey participants indicated “caring for others” as one of their top three stressors (ranging from 0% to 44.4% across the four cities). Differences across ages, genders and ethnicities were not statistically significant. The vast majority (98%) of survey respondents reported they “have happy thoughts about those they help and how they could help” and 94% of respondents “feel a sense of pride in doing their work”. Furthermore, 94% and 95% of respondents, respectively, report that their “work makes them feel satisfied” and that they “make a difference through their work”. This clearly indicates that even through their work can be emotionally draining and stressful, peer workers genuinely care for their community and like to help others.

## Discussion

The results of our study demonstrate that peer workers face multiple sources of stress in their lives and the primary sources of stress are rooted in structural and systemic issues. The most prominent stressors include financial insecurity, lack of respect and recognition at work, housing challenges, lack of support services for PWUD, and constant exposure to trauma at work as well as death of loved ones. It is apparent that for peer workers, personal and professional lives are intimately intertwined; it is difficult to separate one from the other. Thus, while presented separately, the stressors highlighted do not exist independently of each other. Rather, they connect and interact in ways that create a complex, multi-layered structure that can, together, affect peer worker’s mental health and well-being. The stressors highlighted in this paper are reflections of structural issues including current drug laws that criminalize drug use, the illicit drug poisoning crisis, systemic stigma in health and social systems, lack of a living income or wage, supportive housing divestment as well as systems gaps in culturally safe and appropriate services for PWUD [36, 64–67]. Our study found that peer workers’ lived/living experience and association with substance use clouds others’ perception of their identity and that the structural and systemic issues, including stigma and judgement, shape peer workers’ day-to-day experiences, both within the workplace and beyond.

Financial insecurity is one of the most prominent stressors in peer workers’ lives. Several participants attributed their financial insecurity to inequitable pay and job instability. Although peer engagement is recognized as best practice in harm reduction work [62, 63], employment opportunities within organizations that value lived/living experience of substance use are limited [64–67]. Our findings highlight an issue that employers tend to place a higher value on formal education and certification and do not recognize lived/living experience as having equivalent or higher value. This is true for many work settings and is a form of structural violence which perpetuates power imbalances and social inequalities [68].

The notion of valuing academic knowledge over lived/living experience can be linked to the capitalist economic system, which was instituted during the colonial era to elevate rich, white folks who could attend university at the expense of the poor and marginalized folks who were banned from academic spaces [69]. In harm reduction, the majority of peer workers are engaged through precarious or nonstandard work arrangements and paid minimally through honorariums and meagre wages, notably less than that received by other front-line workers and support staff, despite the similarity of their duties [19, 27, 70, 71]. Poor funding for peer worker-led programs and strained budgets is often used to justify inconsistent and low wages for peer workers [72–74]. In reality, as highlighted by others organizations tend to maximize their budgets by “poverty pimping”—leveraging of PWUD’s socio-economic precarity to their own benefit by paying low wages [42]. This socio-economic marginalization and structural inequities that PWUD face in employment contexts is rooted in systemic stigma against PWUD and negative attitudes towards them [72–74]. Precarious working conditions can have severe physical and mental health impacts for different populations [75–77] and can even contribute to burnout [12, 20]. Like previous studies, our findings indicate the need for financial supports for peer workers, including assistance with opening bank accounts, discussing financial barriers and depositing cheques [12]. Organizations must also strive to provide equitable pay for peer workers, based on BC’s peer payment standards [49, 78] as this commitment to providing a living wage and stable employment is essential to reduce stress for peer workers who are at the forefront of overdose response.

Another important stressor discussed by peer workers is lack of respect and recognition from colleagues and other professionals they encounter. Several studies have shown that feeling valued and respected at work are key determinants of an individual’s job satisfaction and perception of good working conditions [79, 80]. This explains why 40% of the survey participants listed “work situation, including working conditions” as one of their top three stressors. Our findings are consistent with other studies, showing that peer workers are not always accepted and respected in the workplace [40, 41, 81, 82]. Previous studies have indicated that the terminology used by support workers to define peer workers is indicative of their negative attitudes towards peer workers, including terms such as “unstable”, “manipulative”, “untrustworthy” and “lacking capacity to participate” [39–41, 81]. The negative attitudes lead to othering of peer workers who are relegated to menial labour, excluded from benefit programs, and shirked of professional development opportunities [20]. In some cases, work settings may require abstinence before PWUD are “employable” [42, 73, 83]. These abstinence-based work cultures tend to perpetuate negative views towards peer workers and continue to oppress an already marginalized population [21]. Our findings highlight that peer workers also feel that they are not respected by other professionals, such as the police and paramedics. Lack of peer worker credibility is a form of structural violence in the sense that it enforces marginalization of PWUD and inhibits them from achieving the same professional recognition as their counterparts without lived/living experience of substance use. In many cases, the negative interactions between PWUD and figures of authority are linked to a culture of surveillance rooted in the colonial endeavour to “control” marginalized populations instead of protecting them [84]. There is an urgent need for immediate implementation of organizational supports for peer workers that account for and begins to mitigate, even in small ways, some of the structural inequities faced by peer workers. Some examples include photograph IDs to increase recognition, official job descriptions and contracts to formalize their roles and create role clarity, skill development opportunities and events where other professionals can get to know them and the work peers do. Increasing recognition of the important work done by peer workers is also necessary to tackle the negative attitudes and stigma towards them. One of our key findings was that peer workers are uncomfortable being referred to as “peers” because it discredits their skills and abilities and defines them by their substance use. Through a voting process during data validation meetings, the term “experiential worker” was selected as an alternative to “peer worker”, but this preference may vary from place to place. Organizations that hire peer workers and researchers who work with this population must endeavour to explore the terminology preferred by the specific group of peer workers and use that preferred language and terms to avoid inadvertently stigmatizing against individuals with lived/living experience of substance use.

Housing challenges is another major stressor for peer workers. According to the Canada Mortgage and Housing Corporation, “acceptable” housing is affordable and should not be more than 30% of one’s income, has enough space to accommodate the family, is in adequate condition that does not require any major repairs [85]. Given peer workers’ descriptions of their living conditions, it is clear that their housing situations are not acceptable. Almost half of the survey participants indicated that they had faced housing challenges in the last 30 days due to their substance use. This highlights how societal sigma and limited income, alongside a highly privatized housing market, hinder an individual’s ability to acquire acceptable housing. Substance use stigma is associated with the unwillingness of landlords to rent to PWUD. This finding was consistent with other studies that suggest marginalized population groups, including PWUD, often face housing challenges [85, 86]. Studies have shown that housing-related issues can significantly contribute to individuals’ stress, affecting their self-esteem and mental health [87]. Our findings support housing as a key determinant of health and an important resource to support employment for peer workers since it affects their productivity and capacity to help others; lack of acceptable housing can be stressful. These results highlight the importance of organizations building relationships with housing providers so that they are better able to refer peer workers and other PWUD to appropriate facilities. Clearly, for organizational strategies to be effective there is a need for increased provincial funding of affordable housing across the province.

Peer workers discussed how lack of access to resources for themselves and other PWUD leads to stress and dissatisfaction. Peer workers see themselves as the bridge connecting PWUD to social services, and this allows them to feel “useful” and gives their lives a meaning [24]. Peer workers often have a profound relationship with the PWUD that they support and consider them “family” [24]. Not being able to refer PWUD to the resources they need, either due to lack of resources or due to lack of skills or credibility to make referrals contributes to a sense of powerlessness. This powerlessness felt by individuals due to their inability to act in a particular manner despite knowing the right course of action is termed “moral distress” [88–91]. Studies have indicated that moral distress can lead to severe health consequences including anxiety, depression, demoralization and workplace alienation [92]. Interventions to reduce moral distress may include skill development opportunities for peer workers in program navigation and operational processes associated with accessing services. Examples of training include familiarizing peer workers with the process of applying for government-issued identification cards or income assistance and training on navigating potentially unpleasant interactions with service providers. Having a designated staff member for building relationships with service providers and giving referrals to PWUD may also be useful for peer workers. Our findings indicate that lack of resources is not the only issue; even when resources do exist, stigma and judgement make these services inaccessible for peer workers. This points to a need for structural and systemic changes to address substance use related harms.

Our study found that peer workers are constantly dealing with the loss of loved ones due to the overdose crisis, which imposes an emotional toll on them. This finding is in line with other studies demonstrating that the overdose crisis has led to considerable grief for PWUD [12, 19, 26, 30]. Exposure to such trauma leads to distress and is termed “compassion fatigue” [93]. A recent study indicated that even a single exposure to a fatal or non-fatal overdose can cause stress, burnout and overdose-related compassion fatigue [31]. For peer workers, the grief and the associated stress is amplified multifold, as they are repeatedly exposed to others’ trauma, coupled with the trauma they face themselves. Despite that, “caring for others” was not among the top stressors. In fact, our findings indicate that the majority of participants take pride in their work and have happy thoughts about those they could help. As discussed in another paper, these feelings seem to be driven by a genuine desire to care for others and the family-like bond they share with PWUD; helping others allows peer workers ascribe meaning to their lives and gives them a sense of purpose [24]. Thus, the meaning peer workers derive from their work continues to motivate them despite frequent hardships, stress and loss that accompany overdose response work [24]. This highlights the need for organizational cultural shifts, which recognize and support peer work. Despite these positive aspects of work that may buffer peer workers from compassion fatigue, the consequences of compassion fatigue cannot be ignored. Studies show that compassion fatigue and burnout can have severe impact on the mental health and productivity of workers [94–96]. Yet, while compassion fatigue has been studied widely among other front-line workers, including nurses [94–98], there is lack of literature on this phenomenon among peer workers. With the onset of COVID-19, reported deaths related to illicit drug overdoses have reached an all-time high [7], causing additional grief and trauma for peer workers as they continue to deal with loss of loved ones. This calls for an urgent need to understand compassion fatigue among peer workers in overdose response settings and design appropriate interventions to tackle this issue. However, it must be noted that designing interventions to tackle compassionate fatigue among peer workers does not address the epidemic of preventable illicit drug overdose deaths in the province. Despite ample evidence to support decriminalization of drug use to prevent deaths associated with illicit drug overdoses [99–101], provincial and federal policies continue to devalue marginalized lives.

For survey results, we tested differences in the perception of certain factors as one of the top three stressors across genders, ages, locations and ethnicities. We found no significant differences across any characteristics, except location. The differences across cities, albeit statistically insignificant for some factors, may be due to high levels of stigma against PWUD in some areas, such as Coquitlam. There are also variances in pay across sites due to the lack of a standardized pay scale for similar jobs between and within regions [42]. Also, different types of sites were represented within the cities; housing agencies in three cities and a peer worker-led harm reduction organization in one city. “Work situation—including working conditions” was found to significantly differ across sites. The site where more than three quarters of the participants reported this to be one of the top three stressors had recently received news of a funding cut around the time the survey was conducted. This may have severely impacted the overall morale of peer workers and the associated stress. On the other hand, the site where only 12.5% of the participants reported “work situation” as one of the top three stressors has a close-knit team of peer workers and managers. Given these site differences, our results suggest that some sites may need to pay extra attention to provide supports for peer workers.

We have highlighted in this paper the stressors that peer workers face, both within their workplace and in their personal lives. Recognition of these stressors and the effects they have on peer workers’ physical and mental health is crucial to inform strategies to better support peer workers. These supports are needed urgently since the onset of COVID-19 has led to a considerable increase in the workload for peer workers due to closures and reduced hours of organizations servicing PWUD [15, 16]. Over time, this increased workload may lead to burnout. As such, there is a critical need to implement supports and strategies that may help to prevent further marginalization of PWUD. Some interventions have been implemented at the four sites included in this paper through the Peer-2-Peer project and will be detailed further in a future paper.

The stressors faced by peer workers are rooted in a range of social, economic, political and environmental factors that systematically disadvantage PWUD [102, 103]. These factors subject PWUD to “structural vulnerability” [36], rendering them vulnerable to both drug and health-related harms [38]. Our findings are consistent with intersectionality theory, which explores how facets of one's social and political identities, including race, gender, disability, substance use, etc., create unique experiences of oppression for each individual [104]. It was not surprising, that with over a quarter of the participants self-reporting as Indigenous, there were some differences observed in stressors across ethnicities, albeit statistically insignificant. Federal initiatives like the Indian Residential School system and the Sixties Scoop have had profound implications for Indigenous family relations [105]. The resulting trauma has been and continues to be passed down inter-generationally, which can be associated with high levels of substance use among Indigenous communities today [105]. This could explain why among Indigenous survey participants “caring for own children” was ranked as one of the top three stressors by 44% of the participants. Given that discrimination and racism are often cited as top stressors for Indigenous communities [106], we expected discrimination to emerge as a top stressor for Indigenous participants. Like other participants, however, “financial factors” was the most prominent stressor for Indigenous participants, and “discrimination” did not rank as one of the top three stressors. However, these results should be interpreted with caution, given that there were only 18 survey participants who self-reported as Indigenous; more research on stressors faced by Indigenous peer workers is needed.

Stigma associated with substance use also remains a major and continuous structural barrier for peer workers [107–109]. This stigma is deeply embedded in the long history of drug prohibition in Canada, which continues to criminalize substance use [110, 111]. This disproportionally burdens PWUD with poverty, homelessness, trauma and health challenges [36, 64–67]. Thus, while different solutions can be offered to support peer workers working in overdose response settings, these programs are impeded by the structural vulnerabilities and continued criminalization of drug use. As such, there is a need to shift our focus to address intersecting social determinants of health like income, education and housing. Unless upstream measures, such as decriminalization, explicitly denounce the negative moral association with substance use, peer workers may never fully feel supported.

Our study has many strengths. Focus group participants had diversity of age, gender, roles, geography and education level. Survey participants included the majority of peer workers at the four sites included in this study (50 out of the 58 peer workers employed during that time, i.e. 86%). The study uses mixed methods which allowed us to compare, contrast and synthesize data from two different sources, leading to robust and comprehensive information. One of the greatest strengths of this study is the use of participatory coding with involvement of PRAs. This prevented implicit bias from academic researchers in the interpretation of results since they may not fully understand the reality of individuals with lived/living experience of substance use.

Despite these strengths, there are limitations as well. One limitation is the lack of generalizability as the study provides data from four metropolitan or large urban centres in BC and the experiences of peer workers in rural settings may be different. However, the sites represent diversity in type, i.e. housing agency versus non-housing and diversity in health regions, as three out of BC’s five health regions were covered. Some limitations were also posed as a result of the data collection methods. Focus group participants may have been hesitant to express their opinions due to fear of judgement from other participants or of jeopardizing their jobs. This concern was mitigated to some extent by keeping the focus groups small, and by ensuring that no managers were present. The focus groups and the surveys were both conducted by peer researchers to promote a power balance and to ensure that participants feel comfortable to provide their honest opinions. In the survey, some questions that required participants to recall their experiences over a 30-day period introduced a potential recall and reflection bias. Some questions were also open to interpretation by the participants, which may have resulted in variability in reporting. Most of the questions in the survey were adapted from validated tools with excellent psychometric properties. However, we do acknowledge that a combination of questions tested for their psychometric properties from different tools does not necessarily guarantee validity of the mixed product. Also, although the survey sample includes most peer workers from each site, the small sample does not allow conclusive inferences about the differences between variables and limits rigour of analyses. Furthermore, the results may not reflect the full range of experiences of peer workers in other contexts. Therefore, more research on the stressors faced by peer workers in various contexts is needed.

## Conclusion

Our study shows that peer workers face multiple sources of stress in their lives. One of the key stressors identified by peer workers was financial insecurity, driven by pay inequity and job instability. Peer workers also lack recognition and respect at work, characterized by lack of job clarity as well as basic resources to put them at an equal footing with support workers who do not have lived/living experience. Additionally, peer workers expressed feelings of powerlessness due to their inability to access or refer individuals to resources. Furthermore, constant exposure to death and trauma results in compassion fatigue among peer workers and takes a toll on their health. Another stressor identified by peer workers was housing challenges, characterized by poor living conditions and difficulty in obtaining safe, affordable housing. The majority of these stressors are rooted in deeper systemic issues such as stigma and criminalization of drug use, which further marginalize an already structurally vulnerable population. With the increase in the workload for peer workers due to COVID-19, these stressors may have been augmented. While our study presents data from a fair sample of peer workers from three out of five health authority regions in BC, more research is needed to understand stressors faced by peer workers employed in overdose response settings across the province.

Given the importance of peer engagement in harm reduction efforts across BC, it is critical to identify the stressors faced by peer workers and implement interventions that ameliorate their negative impacts at an organizational level. However, these stressors may never be fully eradicated until higher-level systemic changes are made, such as decriminalization of substance use. Until this broader shift towards systemic harm reduction is made, organization-level initiatives to mitigate the stressful impacts of peer work can, at most, function as temporary band-aid solutions.

## Data Availability

The datasets used and/or analysed during the current study are available from the corresponding author on reasonable request.

## Appendix

**Table 4:** Frequency distribution of the survey participants and the selection of a factor as one of the “top three stressors” and the association between factors

Characteristics	Total sample	Stressors					
		Financial situation—Yes		Work situation—Yes		Housing—Yes	
	*n* (%)	*n* (%)	*Χ*^*2 **^ of known values	*n* (%)	*Χ*^*2 **^ of known values	*n* (%)	*Χ*^*2 **^ of known values
	(*n* = 50, 100%)	(*n* = 36, 72%)	(*p* value)	(*n* = 20, 40%)	(*p* value)	(*n* = 19, 38%)	(*p* value)
*Gender*			0.267		0.15 (*p* = 0.93)		0.45 (*p* = 0.80)
			(*p* = 0.875)				
Man	27 (54%)	20 (74%)		10 (37.0%)		12 (44.4%)	
Woman	22 (44%)	16 (73%)		10 (45.5%)		7 (31.8%)	
Other	1 (2%)	0 (0%)		0 (0%)		0 (0%)	
*Age*			1.42 (*p* = 0.49)		0.30 (*p* = 0.86)		0.095
							(*p* = 0.95)
40 and under	19 (38%)	11 (57.9%)		6 (31.6%)		6 (31.6%)	
41–50	20 (40%)	16 (80%)		9 (45%)		8 (40%)	
Over 51	9 (18%)	7 (77.8%)		4 (44.4%)		4 (44.4%)	
Unknown	2 (4%)	2 (100%)		1 (50%)		1 (50%)	
*Location*			1.39 (*p* = 0.71)		9.22 (*p* = 0.03)		3.58 (*p* = 0.31)
Coquitlam	9 (18.0%)	8 (88.9%)		7 (77.8%)		6 (66.7%)	
Maple Ridge	8 (16.0%)	7 (87.5%)		1 (12.5%)		1 (12.5%)	
Vancouver	16 (32.0%)	10 (62.5%)		9 (56.3%)		7 (43.8%)	
Victoria	17 (34.0%)	11 (64.7%)		3 (17.6%)		5 (29.4%)	
*Ethnicity*			0.47		0.46		0.13
			(*p* = 0.49)		(*p* = 0.496)		(*p* = 0.72)
Reported Indigenous	16 (32.0%)	10 (62.5%)		8 (50.0%)		5 (31.2%)	
Non-Indigenous	34 (68.0%)	26 (76.5%)		12 (35.2%)		14 (41.1%)	

^*^Yates’ correction

**Table 5:** Summary of relevant survey results

Financial insecurity				
*I feel I am being paid a fair amount for the work I do*				
	Population that responded (*N*) *N* = 49	Agree/strongly agree *N* (% of respondents) *N* = 20 (41%)	Neutral *N* (% of respondents) *N* = 7 (14%)	Disagree/strongly disagree *N* (% of respondents) *N* = 22 (45%)
Coquitlam	9	3 (33%)	1 (11%)	5 (56%)
MR	7	4 (57%)	2 (29%)	1 (14%)
Vancouver	16	4 (25%)	1 (6%)	11 (69%)
Victoria	17	9 (53%)	3 (18%)	5 (29%)

Lack of respect and recognition at work				
*When I do a good job, I get the recognition for it that I should receive*				
	Population that responded (*N*) *N* = 49	Agree/strongly agree *N* (% of respondents) *N* = 25 (50%)	Neutral *N* (% of respondents) *N* = 9 (18%)	Disagree/strongly disagree *N* (% of respondents) *N* = 16 (32%)
Coquitlam	9	1 (11%)	1 (11%)	7 (78%)
MR	8	6 (75%)	1 (13%)	1 (13%)
Vancouver	16	7 (44%)	3 (19%)	6 (38%)
Victoria	17	11 (65%)	4 (24%)	2 (12%)

Poor living conditions				
*In the last 30 days, did you have any housing challenges as a result of your drug use?*				
	Population that responded (*N*) *N* = 48	Always/often *N* (% of respondents) *N* = 11 (23%)	Sometimes *N* (% of respondents) *N* = 12 (25%)	Rarely/never *N* (% of respondents) *N* = 25 (52%)
Coquitlam	9	4 (44%)	2 (22%)	3 (33%)
MR	8	2 (25%)	6 (75%)	0 (0%)
Vancouver	15	1 (7%)	2 (13%)	12 (80%)
Victoria	16	4 (25%)	2 (13%)	10 (63%)

Constant exposure to death and trauma				
*I think that I might have been affected by the traumatic stress of those I help*				
	Population that responded (*N*) *N* = 49	Always/often *N* (% of respondents) *N* = 15 (31%)	Sometimes *N* (% of respondents) *N* = 18 (37%)	Rarely/never *N* (% of respondents) *N* = 16 (32%)
Coquitlam	8	3 (38%)	2 (25%)	3 (38%)
MR	8	4 (50%)	2 (25%)	2 (25%)
Vancouver	16	4 (25%)	8 (50%)	4 (25%)
Victoria	17	4 (24%)	6 (35%)	7 (41%)

*I feel worn out because of my work as an experiential worker*				
	Population that responded (*N*) *N* = 49	Always/often *N* (% of respondents) *N* = 8 (16%)	Sometimes *N* (% of respondents) *N* = 15 (31%)	Rarely/never *N* (% of respondents) *N* = 26 (53%)
Coquitlam	8	1 (13%)	3 (38%)	4 (50%)
MR	8	0 (0%)	2 (25%)	6 (75%)
Vancouver	16	3 (19%)	8 (50%)	5 (31%)
Victoria	17	4 (24%)	2 (12%)	11 (65%)

*I find it difficult to separate my personal life from my life as an experiential worker*				
	Population that responded (*N*) *N* = 47	Always/often *N* (% of respondents) *N* = 8 (17%)	Sometimes *N* (% of respondents) *N* = 14 (30%)	Rarely/never *N* (% of respondents) *N* = 25 (53%)
Coquitlam	8	0 (0%)	1 (13%)	7 (88%)
MR	7	1 (14%)	2 (29%)	4 (57%)
Vancouver	15	3 (20%)	5 (33%)	7 (47%)
Victoria	17	4 (24%)	6 (35%)	7 (41%)

*I have happy thoughts and feelings about those I help and how I could help them*				
	Population that responded (*N*) *N* = 48	Always/often *N* (% of respondents) *N* = 30 (63%)	Sometimes *N* (% of respondents) *N* = 17 (35%)	Rarely/never *N* (% of respondents) *N* = 1 (2%)
Coquitlam	8	3 (38%)	5 (63%)	0 (0%)
MR	8	3 (38%)	5 (63%)	0 (0%)
Vancouver	15	11 (73%)	3 (20%)	1 (7%)
Victoria	17	13 (76%)	4 (24%)	0 (0%)

*I feel a sense of pride in doing my work*				
	Population that responded (*N*) *N* = 50	Agree/strongly agree *N* (% of respondents) *N* = 47 (94%)	Neutral *N* (% of respondents) *N* = 3 (6%)	Disagree/strongly disagree *N* (% of respondents) *N* = 0 (0%)
Coquitlam	9	8 (89%)	1 (11%)	0 (0%)
MR	8	7 (88%)	1 (13%)	0 (0%)
Vancouver	16	15 (94%)	1 (6%)	0 (0%)
Victoria	17	17 (100%)	0 (0%)	0 (0%)

*My work makes me feel satisfied*				
	Population that responded (*N*) *N* = 47	Always/often *N* (% of respondents) *N* = 30 (64%)	Sometimes *N* (% of respondents) *N* = 14 (30%)	Rarely/never *N* (% of respondents) *N* = 3 (6%)
Coquitlam	9	3 (33%)	4 (44%)	2 (22%)
MR	8	4 (50%)	4 (50%)	0 (0%)
Vancouver	14	11 (79%)	3 (21%)	0 (0%)
Victoria	16	12 (75%)	3 (19%)	1 (6%)

*I believe I can make a difference through my work*				
	Population that responded (*N*) *N* = 47	Always/often *N* (% of respondents) *N* = 35 (74%)	Sometimes *N* (% of respondents) *N* = 10 (21%)	Rarely/ never *N* (% of respondents) *N* = 2 (4%)
Coquitlam	8	6 (75%)	2 (25%)	0 (0%)
MR	8	6 (75%)	2 (25%)	0 (0%)
Vancouver	14	8 (57%)	5 (36%)	1 (7%)
Victoria	17	15 (88%)	1 (6%)	1 (6%)

## Abbreviations

BC: British Columbia
BCCDC: British Columbia Centre for Disease Control
COVID-19: Coronavirus Disease of 2019
PRA: Peer research assistant
FG: Focus groups
OPS: Overdose prevention services
PWUD: People who use drugs

## Footnotes

1 A formal or informal site in which overdoses are likely to occur or do occur and there is a response mechanism for overdoses in place.
